# Acoustic Cell Patterning in Hydrogel for Three-Dimensional Cell Network Formation

**DOI:** 10.3390/mi12010003

**Published:** 2020-12-22

**Authors:** Kyo-in Koo, Andreas Lenshof, Le Thi Huong, Thomas Laurell

**Affiliations:** 1 Department of Biomedical Engineering, School of Electrical Engineering, University of Ulsan, Ulsan 44610, Korea; kikoo@ulsan.ac.kr (K.-i.K.); lehuong94alhp@gmail.com (L.T.H.); 2 Department of Biomedical Engineering, Lund University, S-221 00 Lund, Sweden; andreas.lenshof@bme.lth.se

**Keywords:** three-dimensional network structure, fibroblast cells, acoustofluidics, tissue engineering

## Abstract

In the field of engineered organ and drug development, three-dimensional network-structured tissue has been a long-sought goal. This paper presents a direct hydrogel extrusion process exposed to an ultrasound standing wave that aligns fibroblast cells to form a network structure. The frequency-shifted (2 MHz to 4 MHz) ultrasound actuation of a 400-micrometer square-shaped glass capillary that was continuously perfused by fibroblast cells suspended in sodium alginate generated a hydrogel string, with the fibroblasts aligned in single or quadruple streams. In the transition from the one-cell stream to the four-cell streams, the aligned fibroblast cells were continuously interconnected in the form of a branch and a junction. The ultrasound-exposed fibroblast cells displayed over 95% viability up to day 10 in culture medium without any significant difference from the unexposed fibroblast cells. This acoustofluidic method will be further applied to create a vascularized network by replacing fibroblast cells with human umbilical vein endothelial cells.

## 1. Introduction

There is a broad need for three-dimensional (3D) network-structured tissue in engineered organ development and drug development. In particular, engineered 3D microvascular networks are one of the most promising applications of network-structured tissue, where a clinical need is expressed for the treatment of tissue ischemia caused by disease or injury [1,2]. Within engineered tissue, cells should be sufficiently close (within 200 micrometers) to uniformly distribute blood capillary networks in order to prevent the necrotic core formation [3] induced by diffusion-limited oxygen and nutrient supply [4,5,6]. When engineered tissue is implanted, host capillary invasion into the implanted tissue is not sufficiently fast [7]; hence, the functionality and viability of the implanted tissue relies heavily on the already developed vascular networks within the engineered tissue [8].

Using pre-defined polydimethylsiloxane (PDMS) substrates, human umbilical vein endothelial cells (HUVECs) have been seeded to form a network structure and cultured as a vascularized network [9,10,11]. This process is challenging and requires the delicate stacking of individually fabricated layers to accomplish a functional network. Ultrasonic standing waves have been used to pattern HUVECs in parallel sheets in a collagen hydrogel, followed by culturing to form vascularized networks [1,12,13,14]. These methods fabricated a block of hydrogel containing densely aligned HUVECs, which were useful for investigating how HUVECs develop a vascularized network, but not for more recently developed bioprinting techniques; see below. In 2018, Lata et al. demonstrated the use of surface acoustic waves to linearly focus HeLa, PC-12, and MC3T3 cells in a UV-cured hydrogel [15]; however, they neither used this hydrogel as a conduit structure nor as a branched network structure. An alternative method was proposed by Kang et al., using layer-by-layer three-dimensional liquid bioink printing to construct a cell-laden scaffold that embedded hollow network structures [16] (albeit not a vascularized network). Another bio-printing method applying co-axial laminar flows enabled researchers to continuously extrude cell-laden structures of a single-conduit geometry, but not in a network format [8,17,18,19,20,21]. Even though the pioneering work on the above methods inspired studies on vascularized networks, these were not appropriate for the recently developed bio-printing techniques, since they stacked pre-fabricated cell-laden layers with the requirement of a precise alignment. Other bio-printing methods mentioned above could realize channel-embedded scaffolds, but these were not networked to each other, and only demonstrated individual channels without any branches or junctions.

This paper demonstrates a direct extrusion with ultrasound standing waves to manipulate fibroblast cells into a network structure with junctions and branches. Sequential ultrasound frequency alterations from 2 MHz to 4 MHz changed the number of aligned cell streams from one to four in sodium alginate passing through a 400-micrometer square-shaped glass capillary. In the transition from one stream to four streams, the aligned fibroblast cells were continuously rearranged in branched and junction structures. This strategy will be further investigated to create a vascularized network by replacing fibroblast cells with HUVECs.

## 2. Materials and Methods

### 2.1. Device

**Figure 1:**
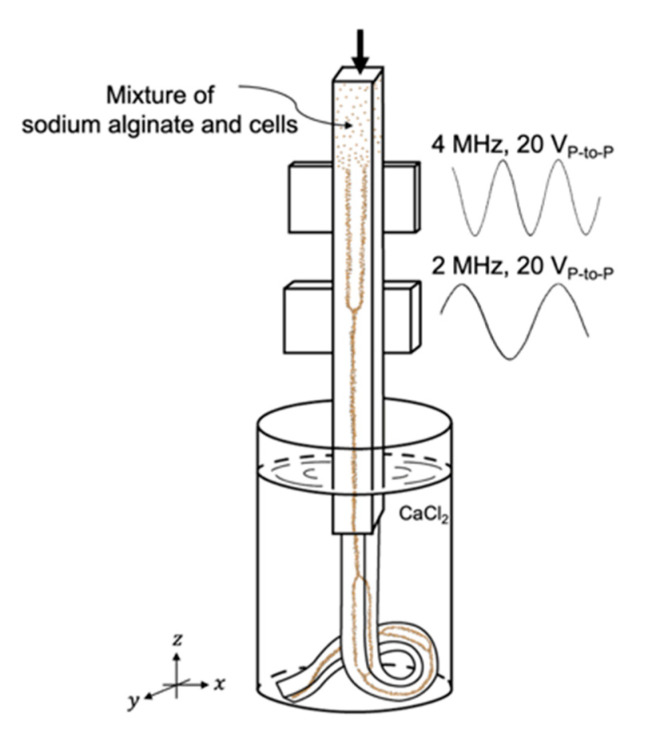
The conceptual sketch of the network generator.

Figure 1 shows a conceptual image of the proposed network generator. A four-hundred-micrometer square-shaped glass capillary (8240, VitroCom, Mountain Lakes, NJ, USA) was glued to a 1 mm thick, 10 × 10 mm ultrasound transducer (F4260302, MEGITT A/S, Kvistgaard, Denmark) for 2 MHz actuation and a 0.5 mm thick 10 × 10 mm ultrasound transducer (F4260570, MEGITT A/S, Kvistgaard, Denmark) for 4 MHz actuation, respectively. One end of the transducer-attached glass capillary was connected to a syringe pump, perfusing the hydrogel precursor mixture material. The other end of the capillary was immersed in a cross-linking solution to gelate the hydrogel precursor mixture.

### 2.2. Cell Culture

Fibroblast cells (NIH/3T3, CRL-1658, cryopreserved) were purchased from a commercial source (ATCC, Manassas, VA, USA). The cell vial was thawed and incubated at 37 °C in a cell incubator, maintaining 5% CO_2_. During expansion, fibroblast cells were supplied with full cell culture media (90% Dulbecco’s Modified Eagle Medium (DMEM) + 10% Fetal Bovine Serum (FBS) + 1% penicillin/streptomycin). The cells were passaged before reaching 80% confluence using 0.05% trypsin/Ethylenediaminetetraaceic acid (EDTA) solution. The cells within passages 8–17 were used in experiments.

### 2.3. Hydrogel Precursor Mixture

As a model system to test the feasibility of the acoustic manipulation of micrometer-sized objects in the alginate hydrogel, inside the capillary, colored polystyrene microparticles were exposed to ultrasound, instead of live cells. Two hundred microliters of 10-micrometer (diameter) polystyrene microparticles with 1.1 × 10^8^ microparticles per milliliter (61946-5ML-F, MERCK, Madison, NJ, USA) were mixed in 3 mL of 0.3% (*w/v*) sodium alginate (W201502, MERCK, Madison, NJ, USA). For the cell manipulation experiment, 200 µL of 1 × 10^7^ cell/mL fibroblast cell suspension was mixed in 3 mL of autoclaved 0.3% (*w/v*) sodium alginate. For cross-linking, the alginate mixture was injected into a beaker of 300 mM CaCl_2_ (746495, MERCK, Madison, NJ, USA).

### 2.4. Network Generation

The cell network-embedded scaffold was generated using a square glass capillary-based bulk acoustic standing wave-controlled network generator (Figure 1). A mixture of fibroblast cells and sodium alginate perfused the glass capillary at 30 µL/min by a syringe pump (WPI SP260P, World Precision Instruments, Sarasota, FL, USA). To align the cells in a single line, the 2 MHz transducer was actuated at 2 MHz, 20 Vpp and, to align the cells in four lines, the 4 MHz transducer was actuated at 20 Vpp. Following the acoustic focusing of the fibroblast cells in the sodium alginate, the aligned cell stream was then injected into CaCl_2_ solution for immediate gelation. Divalent cations (Ca^2+^) in CaCl_2_ solution bound to guluronate blocks of alginate chains in the sodium alginate mixture, then formed junctions with the guluronate blocks of adjacent polymer chains, termed as the eggbox model of cross-linking, resulting in a gel structure [22,23]. The extruded alginate formed a uniform hydrogel string, incorporating the acoustically aligned cells. In order to make a branched network of aligned cells going from one line to four parallel lines and back to one line, the network generator was actuated in the sequence of 2 MHz-4 MHz-2 MHz. When fine tuning the system and checking its feasibility, 10 µm polystyrene microparticles (Sigma-Aldrich, St. Louis, MO, USA) were mixed with sodium alginate and injected instead of live cells.

### 2.5. Cell Staining

After the formation of the 3D alginate cell scaffolds, the CaCl_2_ was removed and the scaffolds were supplied with cell culture media and cultured in a 35 mm cell culture dish at 37 °C, 5% CO_2_. The culture medium was replaced every 2 days. During culturing, on day 1, day 3, day 5 and day 10, the cell scaffolds were stained with LIVE/DEAD staining solution. The staining solution was composed of 0.2% ethidium homodimer-1 (EthD-1 2 mM) in dimethyl sulfoxide (DMSO)/H_2_O 1:4 (*v/v*); 0.05% calcein-acetoxymethyl (calcein-AM 4 mM) in anhydrous DMSO (LIVE/DEAD Viability/Cytotoxicity Kit, for mammalian cells, Molecular Probes, Eugene, OR, USA), and was diluted in 1 × phosphate-buffered saline (PBS) (D8662-500ML, MERCK, Madison, NJ, USA). Before and after staining, the cell scaffolds were lightly washed with 1 × PBS buffer (3 times, 1–3 min per time) to remove media and staining solution.

### 2.6. Microscopic Imaging and Analysis

The cell-stained scaffolds were observed using an IX53 inverted fluorescent microscope (Olympus, Tokyo, Japan) and the images were captured using the CellSens software (Olympus, Tokyo, Japan). For three-dimensional imaging, the FLUOVIEW FV1200 laser scanning confocal microscope (Olympus, Tokyo, Japan) was utilized. The live/dead cell images were analyzed using the ImageJ 1.51h software (National Institutes of Health, Bethesda, MD, USA).

### 2.7. Quantification Method

Cell viability was determined using a ratio calculation between the mean grey value (gv) of 2 color channels (green for live cells and red for dead cells) on a black background. The grey values were obtained from 10× confocal images, which were split into 2 separate color channels using ImageJ 1.51 h. The calculation was carried out according to the following equation [21,24]:

(1) $$Cell\;viability\;=\;\frac{100\;\times \;green\;gv}{green\;gv\;+\;red\;gv}\;$$

### 2.8. Statistical Analysis

The results are presented as the mean ± standard deviation. Five pictures of 2 samples each from 3 experiments respectively, totally 30 pictures were analyzed for the cell viability comparison.

## 3. Results

**Figure 2:**
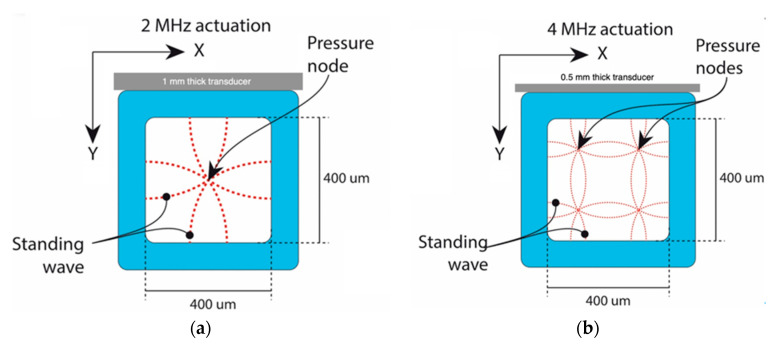
Schematic of the standing wave pattern in the capillary cross-section when actuated (**a**) at 2 MHz, yielding a single pressure node in the center for microparticle or cell focusing, and (**b**) at 4 MHz, providing four pressure nodes, one in each cross-section quadrant for microparticle or cell focusing. The transducer dimensions are not to scale in the drawing.

When applying 2 MHz ultrasound in a 400 µm square-shaped glass capillary, a half-wavelength standing wave was formed in both the x- and the y-direction with the pressure node in the channel center; see Figure 2a. When the frequency was doubled to 4 MHz, the square capillary supported a full wavelength resonance in both the x- and y-directions and hence four pressure nodes were generated in the capillary cross-section, located λ/4 from the channel side walls, Figure 2b.

The acoustic standing wave induces an acoustic radiation force, *F^rad^*, that drives cells and particles in aqueous suspensions towards the acoustic standing wave pressure nodes. The radiation force scales with the cell/particle size, *a*, as well as the acoustic contrast factor, *F*, according to Equation (2), when considering a one-dimensional standing wave [25].

(2) $$F_{x}^{rad}=4\pi \phi \left(\tilde{\kappa },\tilde{\rho }\right)ka^{3}E_{ac}\sin\left(2kx\right)$$

(3) $$E_{ac}=\frac{p_{a}^{2}}{4\rho_{0}c_{0}^{2}}$$

(4) $$\phi \left(\tilde{\kappa },\tilde{\rho }\right)=\frac{1}{3}\left[\frac{5\tilde{\rho }-2}{2\tilde{\rho }+1}-\tilde{\kappa }\right]$$

(5) $$\tilde{\kappa }=\frac{\kappa_{p}}{\kappa_{0}}\;\mathrm{and}\;\tilde{\rho }=\frac{\rho_{p}}{\rho_{0}}$$

where *k* = 2π/λ, and $\phi \left(\tilde{\kappa },\tilde{\rho }\right)$ is the acoustic contrast factor, *E_ac_* the acoustic energy density, *x* the position of the particle in the direction of the channel cross-section (both an x and y direction in this case), *p_a_* the pressure amplitude, *ρ*_0_ and *c*_0_ the density and the speed of sound in the medium, respectively. *E_ac_* is proportional to the square of the voltage applied to the piezoelectric element.

**Figure 3:**
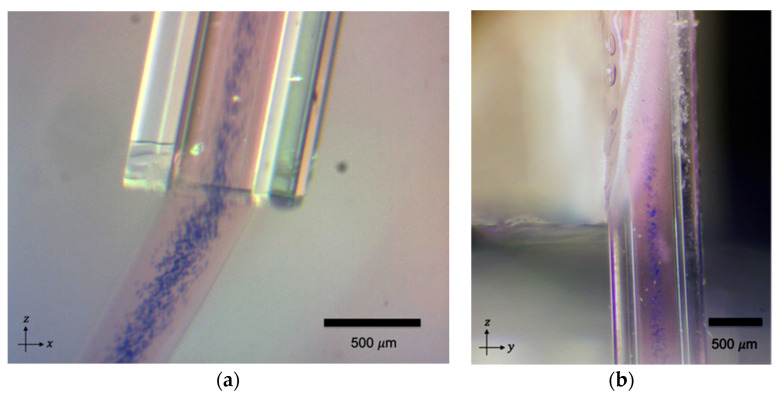
Acoustic focusing of microparticles in the sodium alginate at 2 MHz actuation. (**a**) The view of the focused particles in the y-direction and, (**b**) the view of the focused particles in the x-direction. The blue color shows the polystyrene microparticles. Red food dye was added to the microparticle mixture to give the alginate stream a visual contrast versus the background.

**Figure 4:**
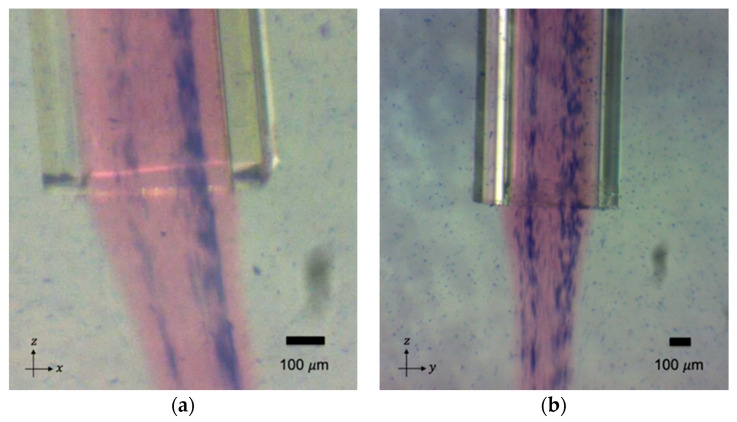
The acoustic focusing of the microparticles in the sodium alginate at the 4 MHz actuation. (**a**) The view of the focused particles in the y-direction and, (**b**) the view of the focused particles in the x-direction. The blue color shows the polystyrene microparticles. Red food dye was added to the microparticle mixture to give the alginate stream a visual contrast versus the background.

The corresponding experimental outcome of the 2 MHz actuation is shown in Figure 3, where the acoustic radiation force focused the microparticles into one stream at the center of the capillary. In the case of the 4 MHz actuation, four parallel lines of microparticles were generated in the sodium alginate, as shown in Figure 4.

**Figure 5:**
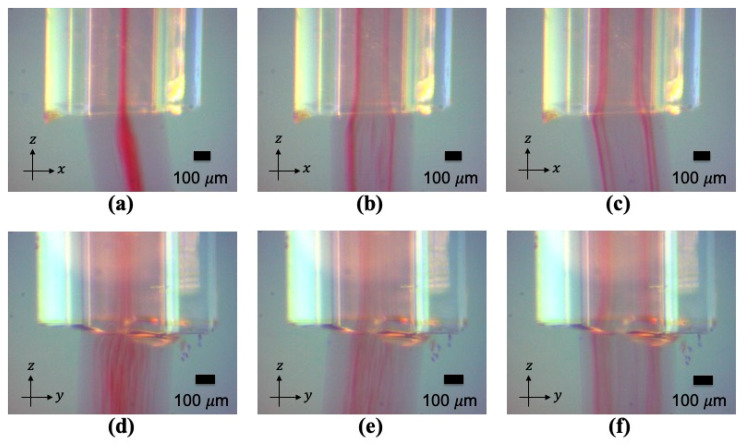
(**a**–**c**) The transient images when switching the actuation from 2 MHz to 4 MHz, imaged in the x-direction. (**d**–**f**) The transient images when switching the actuation from 2 MHz to 4 MHz imaged in the y-direction. The red color shows the polystyrene microparticles.

**Figure 6:**
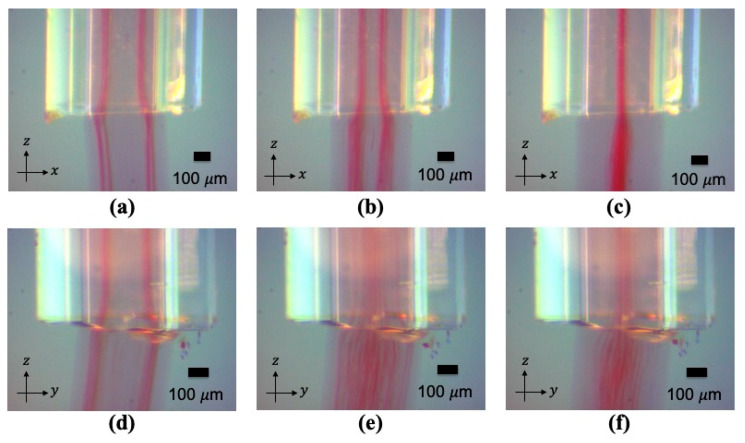
(**a**–**c**) The transient images when switching the actuation from 4 MHz to 2 MHz, imaged in the y-direction. (**d**–**f**) The transient images when switching the actuation from 4 MHz to 2 MHz, imaged in the x-direction. The red color shows the polystyrene microparticles.

**Figure 7:**
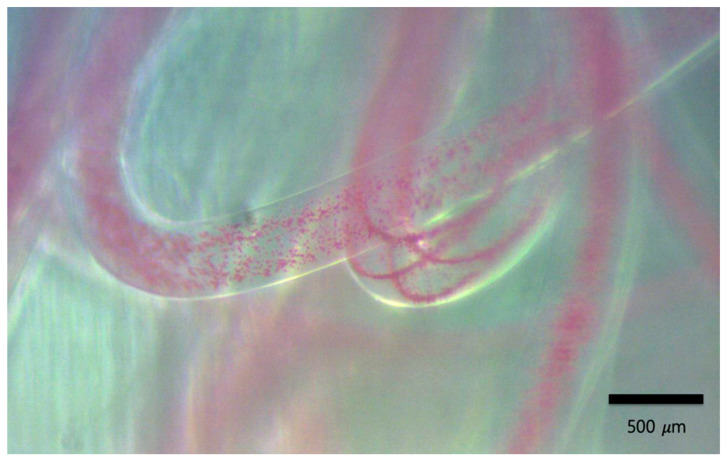
The generated microparticle network in calcium alginate hydrogel. The red color shows the polystyrene microparticles.

When alternating the applied frequency between 4 MHz and 2 MHz, the microparticles were aligned to four streams or one stream according to the selected actuation frequency. The transient steps from one stream to four streams are imaged in Figure 5. The transient course from four streams to one stream are shown in Figure 6. During the transition, the microparticles smoothly moved from one stream to four streams or vice versa. The microparticles were thus aligned as a continuous network structure inside the gelated calcium alginate hydrogel, as shown in Figure 7.

**Figure 8:**
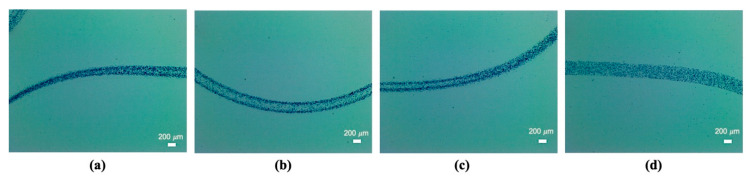
(**a**) The manipulated microparticles in transition from one stream to four streams. (**b**) The aligned microparticles as four streams. (**c**) The manipulated microparticles in transition from four streams to one stream. (**d**) The microparticles unexposed to ultrasound. The blue color shows the polystyrene microparticles.

Figure 8a–c shows the manipulated microparticles as one stream, branching out into to four streams, and again converging into one stream inside the calcium alginate hydrogel. For reference, the microparticle distribution when not exposed to ultrasound is shown in Figure 8d.

**Figure 9:**
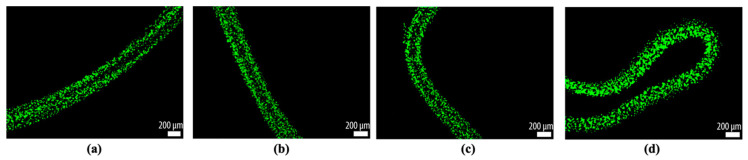
(**a**) The manipulated fibroblast cells in the transition from one stream to four streams at day 3. (**b**) The aligned fibroblast cells as four streams at day 3. (**c**) The manipulated fibroblast cells in the transition from four streams to one stream at day 3. (**d**) The fibroblast cells unexposed to ultrasound at day 3. Live and dead cells were stained green and red, respectively.

After obtaining proof of concept by focusing the microbeads in the sodium alginate into single bands and reconfiguring them via a continuous transition into four parallel bands, we investigated if the same outcome was possible using cells. Figure 9a–c shows fibroblast cells transitioning from a single band of cells to a stream of four bands and, again, switching back to 2 MHz actuation, forming a single band of cells inside the calcium alginate hydrogel. Figure 9d shows the original, non-ultrasound-exposed distribution of the fibroblast cells in a gel string.

**Figure 10:**
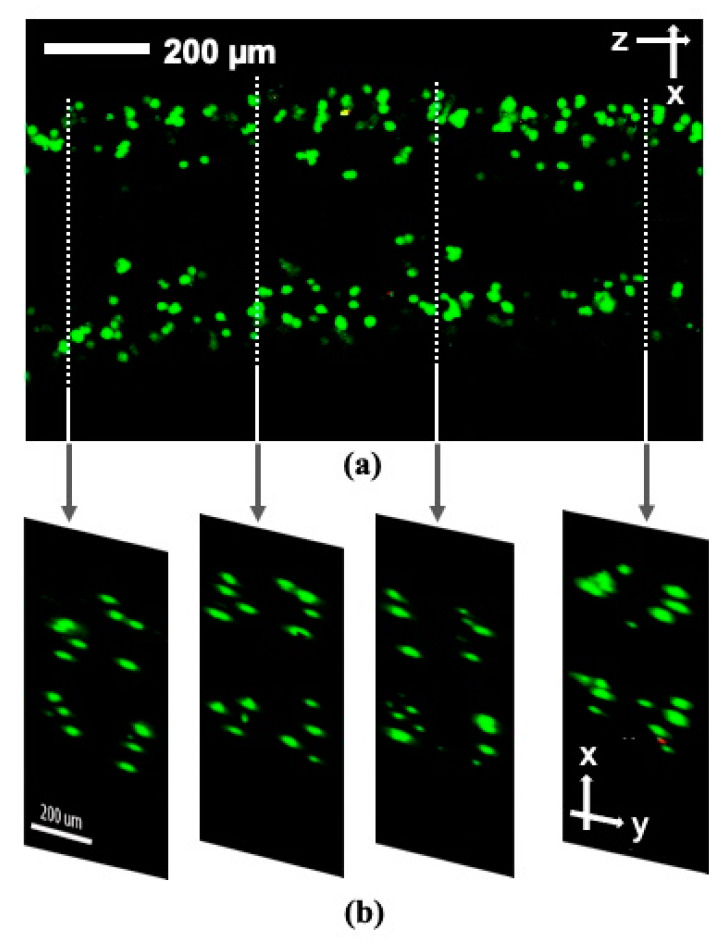
Confocal microscope image of the aligned fibroblast cells as four streams on day 3. The longitudinal view (**a**) and the y-stack image reconstructed cross-sectional views (**b**). Live and dead cells were stained green and red, respectively.

By confocal imaging, the cross-section of the aligned part was reconstructed as shown in Figure 10. The cross-section image was derived from a y-stack confocal reconstruction and not from a gel string cross-section cut, which is why the focus was not perfect. However, four regions of enriched cells were clearly distinguishable, enabling us to infer that the fibroblast cells were aligned as four streams.

**Figure 11:**
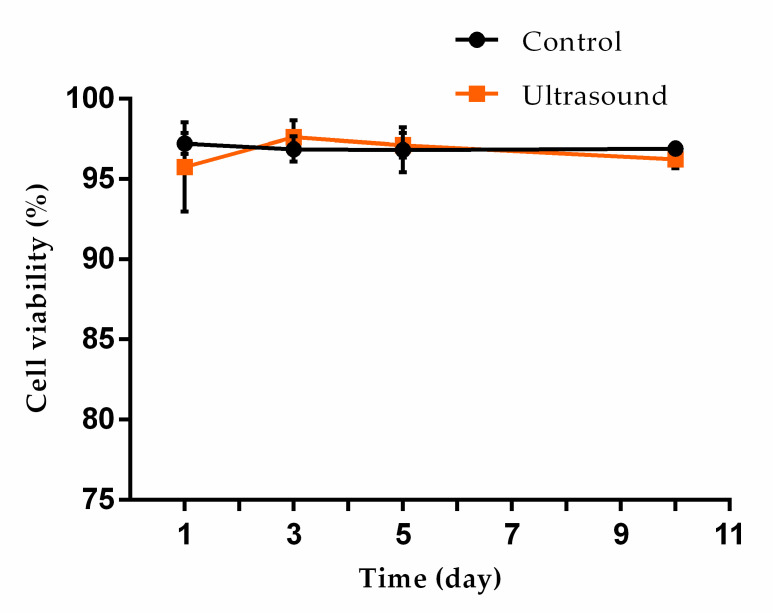
Viability comparison of the ultrasound-exposed fibroblast cells vs. the unexposed control sample.

The viability of the ultrasound-exposed fibroblast cells did not differ significantly from that of the unexposed fibroblast cells, as shown in Figure 11. The viability of the ultrasound-aligned fibroblasts was found to range between 95 and 98% in from day 1 to day 10.

## 4. Discussion

In this study, we demonstrated the continuous extrusion of a fibroblast cell network inside a calcium alginate hydrogel using two ultrasound transducers that led to the two-dimensional acoustic focusing of cells in the extruded hydrogel.

By switching the ultrasound frequency from 2 MHz to 4 MHz, fibroblast cells were continuously rearranged in the hydrogel from one cell stream to four cell streams, like the microparticles in Figure 5. Similarly, when altering the ultrasound frequency from 4 MHz to 2 MHz actuation, fibroblast cells smoothly generated a junction that enabled them to transition from four strings of cells to one string; see Figure 6. By altering the ultrasound frequency, the fibroblast cells could be manipulated to repeatedly form a network structure of cell strings branching out and recombining inside a calcium alginate hydrogel. Compared with the non-ultrasound status shown in Figure 8d and Figure 9d, it was clearly distinguishable that ultrasound enabled the intensive alignment of micrometer-order objects.

The cell viability of the ultrasound-exposed cells and control cells did not differ significantly and was estimated to be 95% after ten-day culturing, as shown in Figure 11. The result was consistent with previous reports that investigated the viability aspects of cells after exposure to ultrasound in acoustophoresis systems [26,27]. Biologists have expressed concern in relation to the use of ultrasound to align cells due to the potential for inducing cell damage similar to the outcome when using common biological laboratory equipment known as “sonication” equipment, which uses low-frequency ultrasound (30~50 kHz) to align or aggregate cells. However, high-frequency ultrasound over 1 MHz in acoustophoresis systems has been reported to be a very gentle and harmless way to manipulate cells, as achieved in this investigation and the studies mentioned above [28].

It is known that the growth of lamellipodia and filopodia in fibroblast cells is not supported by calcium alginate [29], and animal cells do not produce endogenous alginases to enzymatically degrade alginate scaffolds [30]. These restrictions are assumed to induce the round shape of fibroblast cells seen in Figure 9 and Figure 10. From this perspective, Type I collagen could instead be a good candidate scaffold material for the proliferation, migration and differentiation of seeded cells in forthcoming studies [19].

## 5. Conclusions

Using acoustically controlled 3D fibroblast cell patterning, this study demonstrated the potential to arrange live cells into 3D networks within extruded hydrogels. Current studies focus on the manipulation of other cells, such as smooth muscle cells, endothelial cells, and neuronal cells, in Type I collagen for artificial blood vessel network and artificial neuronal network formation.
